# Relational Learning Improves Prediction of Mortality in COVID-19 in the Intensive Care Unit

**DOI:** 10.1109/TBDATA.2020.3048644

**Published:** 2020-12-31

**Authors:** Tingyi Wanyan, Akhil Vaid, Jessica K De Freitas, Sulaiman Somani, Riccardo Miotto, Girish N. Nadkarni, Ariful Azad, Ying Ding, Benjamin S. Glicksberg

**Affiliations:** Hasso Plattner Institute for Digital Health at Mount Sinai, Icahn School of Medicine at Mount Sinai, New York, NY 10029 USA, and the School of Informatics, Computing, and Engineering, Indiana University, Bloomington, IN 47405 USA , and also with the School of Information, University of Texas at Austin, Austin, TX 78712 USA.; Hasso Plattner Institute for Digital Health at Mount Sinai, Icahn School of Medicine at Mount Sinai, New York, NY 10029 USA.; Hasso Plattner Institute for Digital Health at Mount Sinai, Icahn School of Medicine at Mount Sinai, New York, NY 10029 USA, and also with the Department of Genetics and Genomic Sciences, Icahn School of Medicine at Mount Sinai, New York, NY 10029 USA.; Hasso Plattner Institute for Digital Health at Mount Sinai, Icahn School of Medicine at Mount Sinai, New York, NY 10029 USA.; Hasso Plattner Institute for Digital Health at Mount Sinai, Icahn School of Medicine at Mount Sinai, New York, NY 10029 USA, and also with the Department of Genetics and Genomic Sciences, Icahn School of Medicine at Mount Sinai, New York, NY 10029 USA.; Hasso Plattner Institute for Digital Health at Mount Sinai, Icahn School of Medicine at Mount Sinai, New York, NY 10029 USA, and also with the Department of Medicine, Icahn School of Medicine at Mount Sinai, New York, NY 10029 USA.; School of Informatics, Computing, and Engineering, Indiana University, Bloomington, IN 47405 USA.; School of Information, University of Texas at Austin, Austin, TX 78712 USA, and also with the Dell Medical School, University of Texas at Austin, Austin, TX 78712 USA.; Hasso Plattner Institute for Digital Health at Mount Sinai, Icahn School of Medicine at Mount Sinai, New York, NY 10029 USA, and also with the Department of Genetics and Genomic Sciences, Icahn School of Medicine at Mount Sinai, New York, NY 10029 USA.

**Keywords:** Electronic health records, COVID-19, machine learning, deep learning, LSTM, heterogeneous graph model, relational learning, embeddings, ICU, mortality

## Abstract

Traditional Machine Learning (ML) models have had limited success in predicting Coronoavirus-19 (COVID-19) outcomes using Electronic Health Record (EHR) data partially due to not effectively capturing the inter-connectivity patterns between various data modalities. In this work, we propose a novel framework that utilizes relational learning based on a heterogeneous graph model (HGM) for predicting mortality at different time windows in COVID-19 patients within the intensive care unit (ICU). We utilize the EHRs of one of the largest and most diverse patient populations across five hospitals in major health system in New York City. In our model, we use an LSTM for processing time varying patient data and apply our proposed relational learning strategy in the final output layer along with other static features. Here, we replace the traditional softmax layer with a Skip-Gram relational learning strategy to compare the similarity between a patient and outcome embedding representation. We demonstrate that the construction of a HGM can robustly learn the patterns classifying patient representations of outcomes through leveraging patterns within the embeddings of similar patients. Our experimental results show that our relational learning-based HGM model achieves higher area under the receiver operating characteristic curve (auROC) than both comparator models in all prediction time windows, with dramatic improvements to recall.

## 1 Introduction

New York City (NYC) is one of the hardest hit regions of the Coronavirus-19 (COVID19) pandemic with over 200,000 cases at the time of this article. While much has been learned about this disease since its first appearance, there is much not yet understood. It is clear that COVID19 is a complex phenomenon with patients having varied manifestations, long-term outcomes, and systems affected. While it is imperative that we learn as much as we can about this disease as quickly as possible, one of the major issues is that these types of data are difficult to acquire especially in conjunction with patient outcomes. Electronic Health Records (EHR) are a collection of data that relate to patient interactions with a health system, such as lab test results, and have been critical in studies of health effects of COVID19 [1], [2], [3], [4] [5], [6]. Computational data science strategies, especially machine learning (ML), have been deployed to make use of EHR data to aid the clinical process for COVID-19 in key areas such as rapid diagnosis [7], biomarker identification [8], and outcome prediction [9], [10] among many others. While these efforts have met with success, more and more data are being compiled of various modalities that relate to these phenotypes, spanning molecular and clinical features of different types such as cell counts, images, viral load, genomics, among others. In order to truly leverage this massive amount of multi-omic data, standard ML advanced methodologies may not be sufficient. Graph models such as knowledge graphs [11], [12] and graph neural networks [13] are known to better capture the complex interplay between various feature types and can enhance learning within multiple domains [14]. To date, there have not been many studies that have attempted to used graph models for representational learning in the context of COVID19. Ray *et al*. used Graph AutoEncoders to predict possible drug targets [15]; Wang *et al*. built a knowledge graph to identify drugs to repurpose for COVID19 [16]; and Kapoor and Ben et *al.* [17] built graph neural networks on county level US population data to forecast the spread of COVID19.

To our knowledge, there are no studies which leverage the rich EHR patient data from a diverse and highly affected population within a graph model framework for predicting clinically-relevant COVID19 outcomes. In this work, we are the first to develop a novel relational learning strategy using a heterogeneous graph model on EHR data using various clinical features for this purpose. We built this framework to predict mortality at various time frames starting from transfer to an intensive care unit (ICU) using data for over a thousand COVID19-positive patients from five hospitals within NYC. We show that this strategy outperforms baseline models and is extensible for future incorporation of other relevant data types like images and clinical text.

## 2 Preliminaries

In this section, we introduce some preliminary definitions and concepts that are crucial for constructing our relational learning model.

**Definition 1 (Heterogeneous Network).** *A heterogeneous network is defined as a graph* **G** = (*V*, *E*, *T*), *where each node* **v** *and each link* **e** *are represented by their mapping functions to a specific node and relation type ϕ*(*v*) : *V* → *T*_*V*_ *and ϕ*(*e*) : *E* → *T*_*E*_. *Where T*_*V*_ *and T*_*E*_ *denote the sets of node and relation types, and* ∣*T*_*V*_∣ + ∣*T*_*E*_∣ > 2.

**Definition 2 (Heterogeneous Graph Learning).** *Given a heterogeneous network* **G**, *the task of heterogeneous graph learning is to learn a function mapping f* : *V* → *R*^*d*^, *that connects disparate type of nodes into a d* – *dimensional uniform latent representation X* ∈ *R*^∣*V*∣×*d*^, and *d* ⪡ ∣*V*∣, *that are able to capture the structural and semantic relations between them*.

**Definition 3 (One-hop Connectivity).** One-hop connectivity in a heterogeneous network is the local pairwise connection between two consecutive vertices, which directly linked by an edge belongs to a relational type.

**Definition 4 (Two-hop Connectivity).** Two hops connectivity between a pair of vertices in a heterogeneous network is the local connectivity between their one-hop neighborhood; it is determined by whether there is edge connection between their one-hop neighborhood.

### 2.1 Skip-Gram Model

The skip-gram model [18] seeks to maximize the probability of observing the context neighborhood nodes given the center node:

(1) $$\max_{f}\sum_{u\in V}log\;Pr(N_{c}(u)|f(u)).$$

Where *N*_*c*_(*u*) is the neighborhood context nodes of the center node *u*, and *f*(*u*) is the latent representation of *u*.

### 2.2 Heterogeneous Skip-Gram Model

Patient data is heterogeneous, including various type of vertices, such as lab tests, diagnoses, vital signs, and patient demographics. Each of these vertices encodes different information. A Heterogeneous Skip-gram model [19] learns the latent expression of these different type of nodes by maximizing the probability of observing heterogeneous neighborhood given a center node:

(2) $$\max\sum_{u\in V}\sum_{t\in T_{V}}log\;Pr(N_{t}(u)|f(u)).$$

Where *N*_*t*_(*u*) is the heterogeneous neighborhood vertices of center node *u*, and *t* ∈ *T*_*V*_ is the node type.

### 2.3 TransE

The TransE model [20] aims to relate different type of nodes by their relationship type. Specifically, two different types of nodes that are connected by a relationship type would be represented as a triple (head, relation, tail), denoted as (*h*, *l*, *t*). For example, one triple from clinical data could be (*patient*, *diagnosed*, *disease*), where *patient* is the head node, disease is the specific diagnosis attributed to the patient, and the relation between these two vertices is *diagnosed*.

This TransE model leverages the procedure by first projecting different type of nodes with different initial representation dimension into a same latent space (where the dimension of this latent space can be customized). These two different types of projected nodes are linked by a relationship type which is represented as a translation vector in that latent space. Both the projection matrix and the relational translation vector are learnable parameters in a machine learning system.

## 3 Methodology

In this section, we detail the clinical cohort used for this study along with data processing procedures and the architecture our relational learning strategy along with the appropriate baseline comparators.

### 3.1 Clinical Data and Cohort

This study has been approved by the Institutional Review Board at the Icahn School of Medicine at Mount Sinai (IRB- 20-03271). We obtained the Electrical Health Records (EHR) of COVID-19 patients from five hospitals within the Mount Sinai Health System located in New York City. The EHR data collected contains the following patient data:COVID-19 status, Intensive Care Unit (ICU) status, demographics, lab test results, vital signs, comorbid diseases, and outcome (e.g., mortality, discharge). Lab tests and vital signs were measured at multiple time points along the hospital course. We included eight frequently measured vital signs: pulse, respiration rate, pulse oximetry, blood pressure (diastolic and systolic), temperature, height, and weight. We also selected 76 lab tests that were both commonly measured and relevant to COVID-19. For the static features, we included age, gender, and race as demographics and 12 comorbid diseases: atrial fibrillation, asthma, coronary artery disease, cancer, chronic kidney disease, chronic obstructive pulmonary disease, diabetes mellitus, health failure, hypertension, stroke, alcoholism, and liver disease. Comorbid diseases were considered from their presence at admission to the hospital and defined via ICD9/10-CM codes collapsed by Phecode (https://phewascatalog.org/phecodes; see https://github.com/Glicksberg-Lab/Wanyan-TBD-2020 for full mappings used). Please also refer to Appendix A, which can be found on the Computer Society Digital Library at http://doi.ieeecomputersociety.org/10.1109/TBDATA.2020.3048644, for more details regarding these variables.

For this study, we selected patients who were: COVID-19 positive (defined by a positive reverse transcriptase polymerase chain reaction assay of a nasopharyngeal swab), admitted to the hospital, and transferred to the ICU. Furthermore, we only considered patients with a completed stay (i.e., those that either died or were discharged. These filtering steps resulted in 1,269 patients, 328 (25.9 percent) of which died. The cohort breakdown is as follows: mean age was 62.3 ±16.2 std; 784 (61.8 percent) males and 485 (38.2 percent) females; and 341 (26.9 percent) African American, 283 (22.3 percent) White, 50 (3.9 percent) Asian, 537 (42.3 percent) Other, and 58 (4.8 percent) Unknown race.

### 3.2 Data Pre-Processing

The lab test and vital sign features were pre-processed to deal with erro-neous values and varying scales. For each of these features, we discarded measurements that were above 99.5 and below 0.5 percentile from all training values. We then computed the mean and standard deviation of each feature and normalized the data by first subtracting the mean value from the measured value then dividing it by the standard deviation. In this way, we converted different feature values into same scale.

The initial feature input for vital signal at every time point along the ICU stay is a vector *X*_*v*_ ∈ *R*^8^ representing those eight features (blood pressure is separated into its two constituent components: *systolic* and *diastolic*. Every entry of the vector records the normalized numerical value for a specific value measurement at a specific time. Similarly, the initial feature input for lab tests is a vector *X*_*l*_ ∈ *R*^76^ representing the 76 lab test features at a specific time point.

For the static features, demographics consists of three categories. For *Age*, we record the actual numerical value for each patient and normalized it as the same procedure for vital signs and lab tests. For *Gender*, we use a two dimensional one hot vector to represent male or female. For *Race*, we use a five dimensional one hot vector to represent the different race groupings. We form a vector *X*_*d*_ ∈ *R*^8^ to represent the demographic feature input. For the disease comorbidty features, we represent it by a 12 dimension one-hot vector, and concatenate it to the demographics vectors and finally forms 20 dimensional vectors.

### 3.3 Using an LSTM Model to Process Longitudinal Data

We applied an LSTM model to deal with the time sequences of multiple vital sign and lab test data [21]. These data are broken up by time steps (see below). The utilized mathematical for LSTM model is as follows:

$$f_{t}=\sigma (W_{f}\cdot [h_{t-1},x_{t}]+b_{f})\;i_{t}=\sigma (W_{i}\cdot [h_{t-1},x_{t}]+b_{i})\;{\hat{c}}_{t}=tanh(W_{c}\cdot [h_{t-1},x_{t}]+b_{c})\;c_{t}=f_{t}*c_{t-1}+i_{i}*{\hat{c}}_{t}\;o_{t}=\sigma (W_{o}[h_{t-1},x_{t}]+b_{o})\;h_{t}=o_{t}*tanh(c_{t}).$$

Where *x*_*t*_ is the input feature values at time step *t*, *f*_*t*_ is the forget gate, *c*_*t*_ is the cell state, *h*_*t*_ is the hidden state, ${\hat{c}}_{t}$ is the intermediate cell state unit.

We use this LSTM model to connect patient input features to the proposed relational learning layer. Since demographic and comorbid disease features do not change during the visit timeline they are not added to LSTM model. Instead, the input to LSTM model is the concatenation of vital signal and lab test features, which forms a *X* ∈ *R*^83^ input vector. For each time step (two hours) in LSTM model, we create a time window, every event(*measurement for vital signal and lab test*) happened within this time period is recorded in the concatenated 83 dimensional vector as the feature input in that time step. For missing measurement values, we pad zeros to those entries.

For demographic and comorbid disease input features, we apply a separate fully connected layer to map the demographic input vector to a latent embedding representation, then concatenate this latent representation with the hidden representation from the LSTM model output at the final time step. We then use this concatenated hidden representation as the final patient representation *X*_*p*_ ∈ *R*^*m*^ for relational learning. We display this procedure in Fig. 2.

### 3.4 Connecting the Hidden Representation From LSTM With a Relational Learning Model

We create a heterogeneous graph model in the final step after generating the hidden embedding vector from the LSTM model. This model includes two types of nodes: *Outcome* and *Patient*, where the *Outcome* node type contains *Death* and *Discharge*. We connected patients who did not survive to the *Death* node and those who were eventually discharged to the *Discharge* node. We specify a relationship type *Outcome* to connect *Patient* node to *Death* and *Discharge* node. With this procedure, we can create a heterogeneous graph which can be represented as the following triples:

$$Patient\overset{Outcome}{\to }Death\;:\;(Patient,outcome,Death)\;Patient\overset{Outcome}{\to }Discharge\;:\;(Patient,outcome,Discharge).$$

The *Patient* node embedding representation *X*_*p*_ is the concatenated hidden embedding representation introduced in Section 3.3. The initial *Death* and *Discharge* nodes are represented as two dimensional one-hot vectors separately, where *X*_*d*_ = [0, 1] represents *Death*, and *X*_*c*_ = [1, 0] represents *Discharge*.

**Fig. 1.**
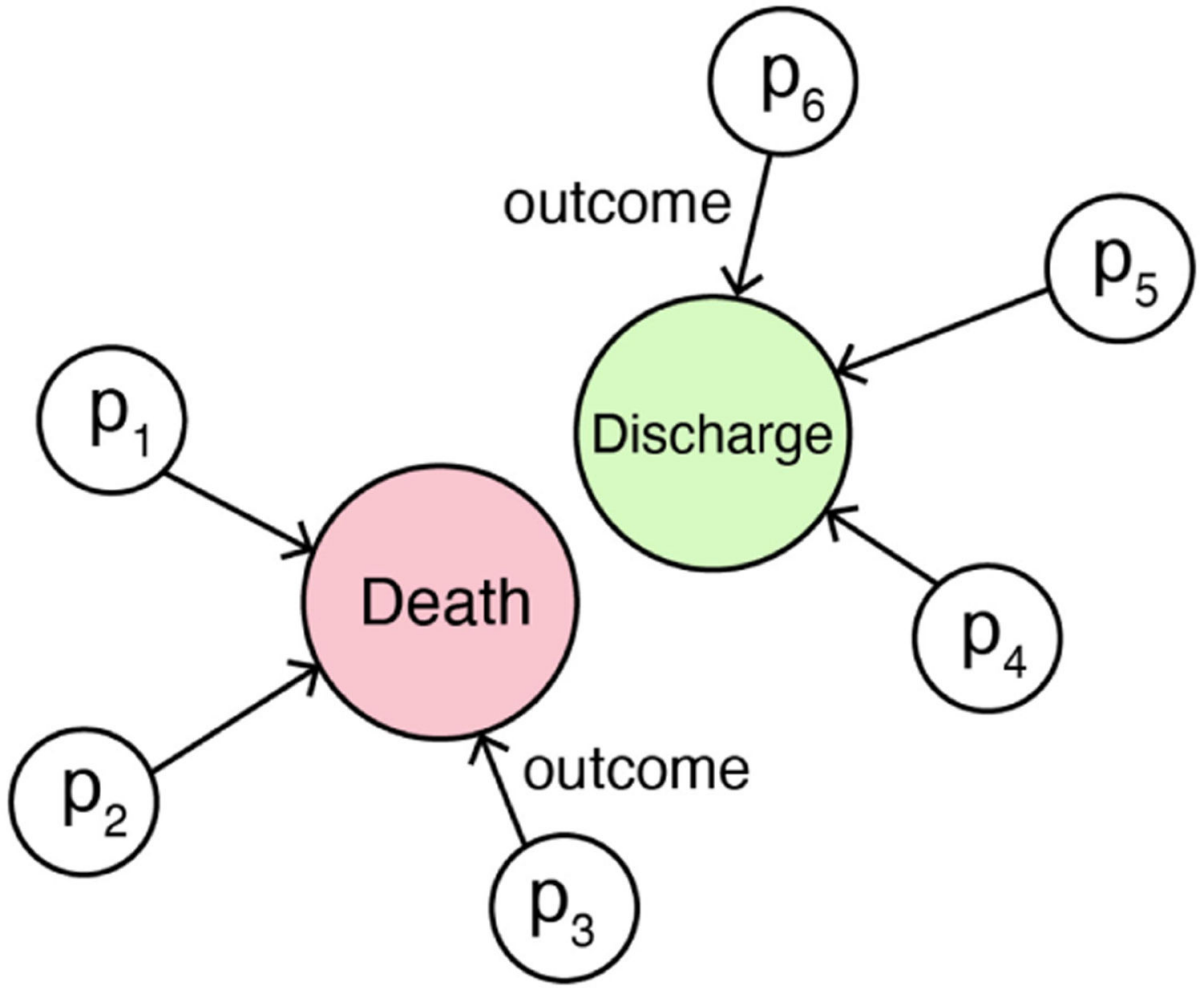
A HGM constructed to connect patients to death or discharge nodes.

With the type of relationship *Outcome*, we can construct a heterogeneous graph integrating all patient data from our cohort (Fig. 1). Both *Death* and *Discharge* nodes have connections with all *Patient* nodes based on their outcome. Through this operation, patterns can be learned between patients that have the same outcomes.

### 3.5 Embedding the Heterogeneous Graph Model Into a Latent Space

**Fig. 2.**
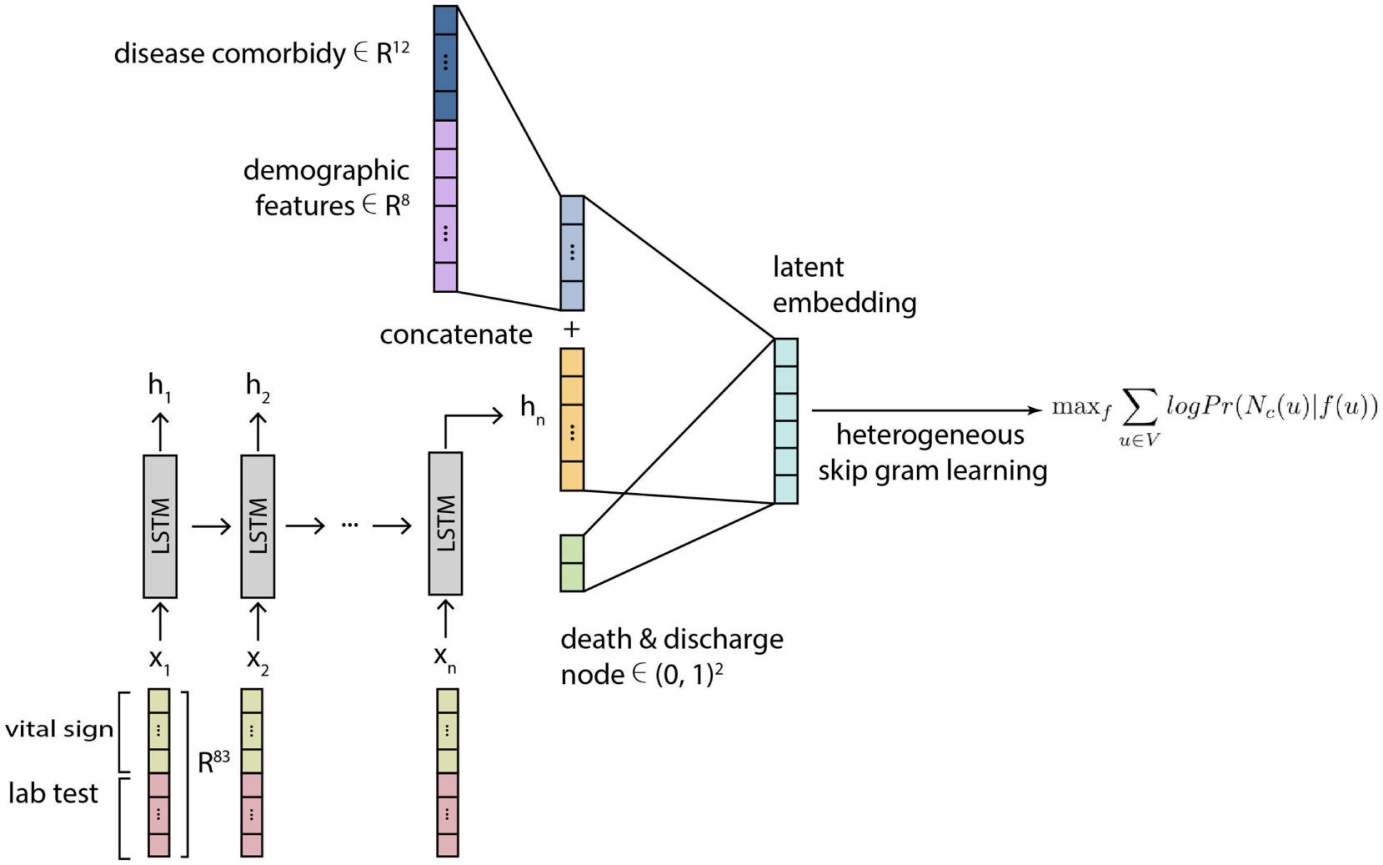
Schematic figure for the LSTM + HGM architecture. Multi-time-point features, specifically vital signs and lab tests, are encoded within an LSTM model while static features (demographics and disease comorbidities) are concatenated within the final layer. Relational learning is then applied to an HGM.

Nodes from the constructed HGM can be embedded into a shared latent space using the TransE [20] method (Fig. 2 Right Side). This model uses a set of 1) projection matrices and 2) relation vectors. After initialization, projections and translations can be optimized end-to-end (see section 3.6).

Heterogeneous graph model nodes *X*_*p*_, *X*_*d*_, *X*_*c*_ are projected into a shared latent space with trainable projection matrices *W*_*p*_, *W*_*m*_ using these nonlinear mappings:

(3) $$C_{p}=\sigma (W_{p}\cdot X_{p})\;C_{d}^{*}=\sigma (W_{m}\cdot X_{d})\;C_{c}^{*}=\sigma (W_{m}\cdot X_{c}).$$

Where *σ* is a non-linear activation function, *W*_*p*_ ∈ *R*^*k*×*m*^, *W*_*m*_ ∈ *R*^*k*×2^ are the projection matrix. *k* is the projected dimension, *m* is the dimension of patient embedding representation. *C*_*p*_, $C_{i}^{*}$, $C_{d}^{*}$ are the projected latent representations of each type of node. We use the same projection matrix *W*_*m*_ to project both *Death* and *Discharge* nodes because they both belong to Outcome node type. Despite the EHR-space using different dimensions for different node types *X*_*p*_, *X*_*d*_, *X*_*c*_, all nodes types are projected into the same dimension latent space.

Then we apply translation operations(subtract a relational vector *R*_*m*_ from $C_{d}^{*}$ and $C_{c}^{*}$) to semantically translate the projected embedding representation $C_{d}^{*}$ and $C_{c}^{*}$ into the same latent space of patient embedding *C*_*p*_:

(4) $$C_{d}=C_{d}^{*}-R_{m}\;C_{c}=C_{c}^{*}-R_{m}$$

Where *R*_*m*_ is the relation vectors representing relation type *Outcome* connecting patients to *Death* and *Discharge* nodes, respectively. Eq. (4) ensures to use translation relation *R*_*m*_ to further translate the projected outcome node latent representation $C_{d}^{*}$ and $C_{c}^{*}$ into the same latent space of patient *C*_*p*_, so that we can apply similarity comparisons(inner product) between these two representations in that latent space. Therefore, *C*_*d*_ and *C*_*c*_ is the eventual projected medical outcome representation that lies in the same space with *C*_*p*_.

### 3.6 Optimizing the Heterogeneous Graph Model Embedding

With the projection and translation operations we can convert different types of nodes into the same latent space. We then tune these parameterized transforms to increase the proximity between those embedding points whose corresponding graph nodes are often connected. Specifically, we apply the relational learning strategy: Heterogeneous Skip-gram optimization [19] using the optimization model:

(5) $$\max\sum_{u\in V}\sum_{t\in T_{V}}logPr(N_{t}(u)|f(u)).$$

Where *N*_*t*_(*u*) is the heterogeneous neighborhood vertices of center node *u*, and *t* ∈ *T*_*V*_ is the node type. Here, we effectively learn node embeddings by maximizing the probability of correctly predicting the center *patient* node’s associated *Outcome* node. The prediction probability is modeled as follow:

(6) $$Pr(c_{t}|f(u))=\frac{e^{{\vec{c}}_{t}\cdot \vec{u}}}{Z_{u}}.$$

Where $\vec{u}$ is the latent representation of a center patient node *u*, ${\vec{c}}_{t}$ is the latent representation of the one-hop neighbor *Outcome* node and two-hops neighbors *Patient* nodes of *u*, and ${\vec{c}}_{t}\cdot \vec{u}$ is the inner product of the two embedding vectors representing their similarity. *Z*_*u*_ is the normalization term $Z_{u}=\sum_{v\in V}e^{{\vec{v}}_{t}\cdot \vec{u}}$. Where *Z*_*u*_ integrate over all vertices. Therefore, Eq. (5) could be simplified to:

(7) $$L_{s}=-\sum_{t\in T}\sum_{u\in V}\left[\sum_{c_{t}\in N_{t}(u)}{\vec{c}}_{t}\cdot \vec{u}-logZ_{u}\right]$$

Numerical computation of *Z*_*u*_ is intractable for very large graphs with millions of nodes. So we adopt negative sampling strategy [18] to approximate the normalization factor, making the optimization function as such:

(8) Ls=−∑t∈T∑u∈V[∑ct∈Nt(u)logσ(c→t⋅u→)][+∑j=1KEcj∼Pv(cj)logσ(−c→j⋅u→)],

where $\sigma (x)=\frac{1}{1+\text{exp}(-x)}$, K is the number of negative samples. *P*_*v*_(*c*_*j*_) is the negative sampling distribution. Eq. (8) is the final objective function we are using for heterogeneous graph learning.

For training the heterogeneous graph model, we first pick the one-hop neighborhood *Outcome* node (either *Death* or *Discharge* node) that connects to the center *Patient* node, then we pick the two-hop connectivity neighborhood *Patient* nodes that connects to the same *Outcome* node. Note that we connect similar patients (both died or discharged) through this operation, so we can use relational learning (heterogeneous skip-gram learning) to propagate information between similar patients, and update the final patient embedding.

Specifically, for one center *Patient* node in training, we first pick the *Outcome* node the patient connects to (i.e., *Death* or a *Discharge*), From the selected *Outcome* node, we then uniformly sample another ten *Patient* nodes that are two-hops connected to the center *Patient* node. In this way, we can connect the center patient node with highly similar other *Patient* nodes via their shared outcome status. For negative sampling [18], we first pick the *Outcome* node which the patient does not connect to, then we perform uniform sampling through all *Patient* nodes that do not have two-hops connections with the center training patient node. Then we project these different nodes into same latent space through TransE model: after unifying the embeddings for different node types, each concept is represented as a point in a euclidean space. In this space we can measure the similarity between any two points by the angle between vectors between them and the origin.

## 4 Experiment

In this section, we describe the process of performing all experiments, including training and testing procedures and baseline comparator methods. We used 70 percent from our cohort as training set and 30 percent as testing set. For all models, we predict the event outcome at varying lengths of time (time windows) prior to the event, specifically 6, 12, 24, and 48 hours. These time windows are broken down by two hour time steps as previously described.

### 4.1 Baselines

#### MLP(Multi Layer Perceptron)

The first baseline we use is a shallow neural network with a fully connected layer that connects the features with the logit output. We then apply a softmax layer on top of logit output to predict patient mortality. Since this model does not utilize time-varying features, we take the mean value of the vital sign and lab test features along the entire patient’s time line, and concatenate these with demographic and comorbidity features.

#### LSTM

The second baseline we are comparing is a LSTM model with softmax layer as the final classification layer. Note that the LSTM baseline method is the same way in which our relational learning model deals with multiple time points of vital and lab features. The difference is that in our LSTM + HGM, we use a relational learning layer at the end instead of a softmax layer.

### 4.2 Experimental Design

Our cohort contained 1,269 patients, with 328 of eventually dying. As such, we have much more negative labels than positive labels. A more robust system should be tolerant of this unbalanced class label and be able to predict the minority group labels. As another challenge, patient data tend to vary in terms of length of stay. For example, a discharge event could happen as early as a few days or over a month from transfer to ICU. So when we train the system to assess risk of death for a certain period given a previous training time window, events may not always occur in these time frames leading to spare outcomes. As such, we create different time windows prior to the event (i.e., whether a patient died or was discharged), and use this time window to do prediction on either the training set or the test set.

We record f1, accuracy, auroc, preciton and recall scores both for training and test sets to observe different models performance in various evaluation metrics.

## 5 Results

In Table 1, we observe that using a relational learning model consistently outperforms all baseline methods with respect to f1, accuracy (percentage correctly labeled), auroc score, and recall scores. The LSTM + HGM f1 scores are consistently 30 to 40 percent higher than both MLP and LSTM models. This increase is due primarily to the huge increase in performance within recall. The low testing recall and f1 score for both MLP and LSTM shows the deficiency of machine learning models that apply per-data training via a final softmax layer. This procedure updates system parameters in one training iteration to be independent of other similarly relevant data. So when the training data class label is very unbalanced, especially in a situation seen in our COVID19 ICU cohort, these models tend to overfit to the majority group labels. Therefore, even though we observe high precision score from LSTM and MLP model, their f1 score and recall are considerably low, indicating an inability to capture the risk for death for all patients who will do so.

In contrast, our relational learning layer seen in the LSTM + HGM model propagates information from similar patients who both connect to the same outcome node. The Heterogeneous Skip-Gram learns scenarios that maximize the similarity between patients that connect to common event (i.e., Death or Discharge) and meanwhile minimizes the similarity between patients that do not have connections through common event. This is a good strategy in dealing with situations when we do not have balanced classes. Similar patients’ pattern information can be shared and learned via embeddings in addition to their class labels, which optimizes the learning ability to best find the patterns that discriminate between different groups and distinguish them.

**Fig. 3.**
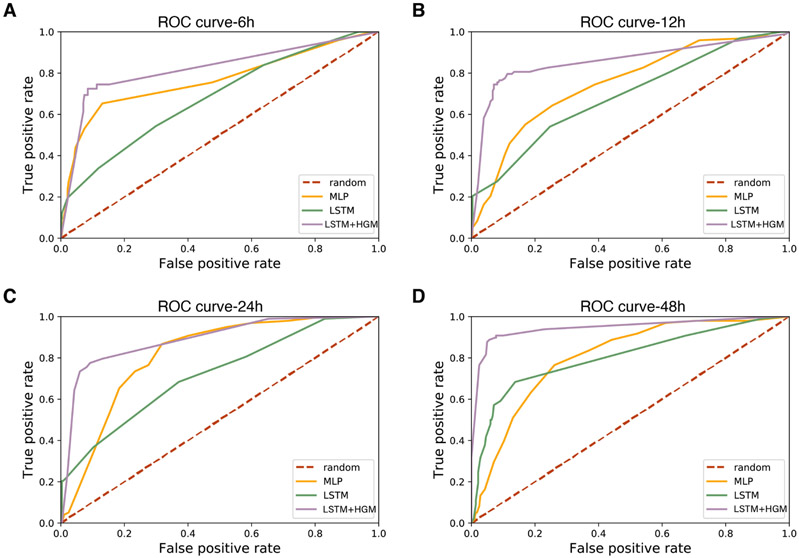
Testing data ROC curve measurements for different models on different prior time windows

The ROC curve (Fig. 3) shows the effectiveness of this approach. Through using all thresholds as classification criteria, the relational learning model outperforms both baselines to a large degree. It was interesting to note that a simple MLP model which averages multiple time points of data performs well in short prediction time windows, but the performance decrease as the prediction time window duration increases from the event. In contrast, the LSTM and LSTM + HGM approaches see improvements in performance as more longitudinal data is provided to the model.

The precision score from our relational learning model is relatively lower compared to baseline LSTM because we hypothesize the latter is more overfit to the major class group, leading to a low false positive rate, while relational learning model predicts more positive labels overall since it better balances the minor class labels through information sharing. Despite the somewhat lower precision scores, this strategy ultimately has much improved overall accuracy and f1 score.

**Fig. 4.**
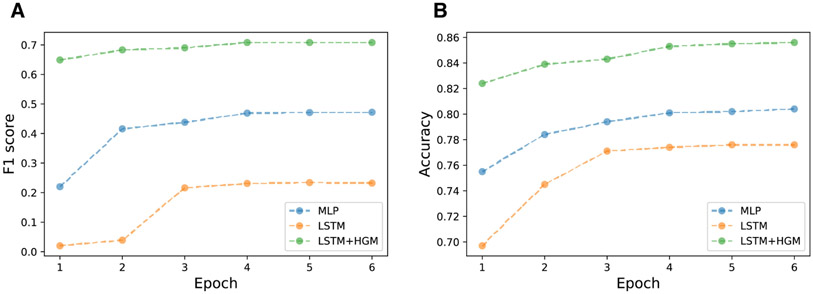
f1 & accuracy vs training epoch for 6 hour prediction time window.

The training f1 score& accuracy vs epoch plot (Fig. 4) shows that the relation learning model converges to fixed accuracy and f1 score that is overall higher and did so much quicker, meaning that the learning strategy is more efficient so that it can quickly reach optimal point.

## 6 Discussion

The complex nature and manifestation profiles of COVID-19 suggests the need for machine learning algorithms to appropriately model the heterogeneous types of patient data for more accurate predictions. In this work, we sought to predict mortality for COVID-19 postive patients that were transferred to the ICU. We hypothesized that a relational learning strategy that also takes into account the varying and dynamic nature of frequently measured vital signs and lab test measures would have optimal performance. As such, we developed a framework that incorporates an LSTM to model time varying features as well as a relational learning layer via a HGM. We compared this framework to two relevant baseline machine learning comparator models, specifically a shallow MLP and an LSTM with a softmax layer. We performed experiments on different time windows (e.g., 6, 12, 24, and 48 hours) leading up to the outcome (i.e., death or discharge, see Table 1, as a way to assess the different model frameworks ability to predict outcomes using different amounts of data throughout the time course of the hospitalization. We performed this experiment on a large and diverse cohort from the Mount Sinai Health System, totalling 1,269 patients from five affiliated hospitals.

In our study there a there were more negative labels (n = 941) than positive (n = 328) resulting in a class imbalance. Furthermore, there were challenges in the fact that different time varying features had different frequencies and compelteness across time. Regardless, we developed an experimental strategy which lead to effective utilization of LSTM architecture. Interestingly, the MLP model was able to achieve decent performance at short time windows prior to the event but its performance dropped as greater time lengths were added. On the other hand, the performance of the LSTM models increased when more longitudinal data was added. Our biggest finding, however, was the huge improvements in performance by adding a relational learning HGM layer instead of the traditional softmax to the LSTM models. This performance was driven by large gains in recall between the two.

Our study had several limitations which need to be addressed. First, while our cohort is one of the largest and most diverse, it does consist of patients only within the New York City region and therefore our model may not be generalizeable to the outside population. Second, we predicted mortality at time windows leading up to the event and not from onset of transfer. This was based on initial evaluation of this framework as a proof-of-concept which future work will extend upon. Third, there are other key clinical variables that may be of value to the model that were not added based on lack of availability. Fourth, our models were not assessed using cross-validation. Fifth, while we believe we have selected appropriate baseline models to compare our proposed method against, there are many other machine learning models as well as ways to model time series that our framework should be measured against in future work.

In our future work, we plan to refine our framework by modeling the HGM dynamically over time. We will also use this framework for predicting other highly relevant clinical outcomes, such as development of acute kidney injury. We also plan on incorporating other patient data modalities within the relational learning framework, including electrocardiogram signals in the form of physiological wave form data, imaging features from x-rays, and genomics. This framework will also be applied to predict outcomes of other diseases.

In conclusion, we believe this work is one of the first to demonstrate the utility of relational learning and heterogeneous graph models in predicting COVID-19 mortality for those within an ICU. The relational learning strategy employed allowed for better modeling of the various types of patient data which resulted in superior performance over relevant baselines. With further testing and improvements, we hope to be able to test this framework within hospital operations with the eventual goal of aiding clinical practitioners’ fight of this pandemic.

## Supplementary Material

supp1-3048644

## Figures and Tables

**TABLE 1:** Evaluation of Different Algorithms on Predicting Risk of Mortality

Lead Time Window	Training set					Test set				
	f1	accuracy	auroc	precision	recall	f1	accuracy	auroc	precision	recall
MLP										
6h	0.438	0.784	0.786	0.720	0.295	0.487	0.803	0.766	0.814	0.368
12h	0.435	0.757	0.797	0.719	0.212	0.446	0.748	0.746	0.603	0.322
24h	0.314	0.729	0.809	0.885	0.191	0.269	0.706	0.762	0.705	0.167
48h	0.422	0.759	0.808	0.887	0.214	0.295	0.729	0.775	0.804	0.275
LSTM(softmax)										
6h	0.208	0.776	0.681	0.897	0.117	0.205	0.779	0.669	0.882	0.132
12h	0.251	0.764	0.709	0.867	0.147	0.322	0.787	0.689	0.947	0.193
24h	0.248	0.770	0.701	0.861	0.144	0.336	0.787	0.718	0.924	0.204
48h	0.610	0.832	0.806	0.773	0.504	0.585	0.821	0.790	0.737	0.489
LSTM + HGM										
6h	0.741	0.871	0.825	0.753	0.743	0.690	0.843	0.821	0.718	0.684
12h	0.801	0.901	0.866	0.832	0.774	0.779	0.887	0.857	0.784	0.776
24h	0.818	0.873	0.861	0.886	0.778	0.781	0.877	0.824	0.778	0.785
48h	0.875	0.935	0.922	0.877	0.874	0.748	0.847	0.879	0.727	0.837

Lead Time Window refers to the amount of time utilized prior to outcome.
