# Supplementary material for: Relational Learning Improves Prediction of Mortality in COVID-19 in the Intensive Care Unit
Source: IEEE Trans Big Data. Author manuscript; Available in PMC 2021 Mar 24. (PMC7990133; doi:10.1109/TBDATA.2020.3048644)
Supplement: Supplementary file 1 [file NIHMS1678826-supplement-supp1-3048644.docx]

**Supplementary Materials**

**List of Lab Test Features**

Albumin, Alkaline Phosphatase, Alanine Aminotransferase, Amylase, Anion Gap, Partial Thromboplastin Time, Aspartate transaminase, Atypical Lymphocytes, Band Cells, Basophil %, Basophil Count, Direct Bilirubin, Total Bilirubin, Myeloblasts, Brain Natriuretic Peptide, Blood Urea Nitrogen, C-reactive protein, Ionized Calcium (Arterial), Ionized Calcium, Calcium, Chloride, Creatine phosphokinase, Creatine kinase-MB, Bicarbonate, Creatinine, D-dimer, Eosinophil %, Eosinophil, Ferritin, Fibrogen, Glucose, Glucose (Arterial), Bicarbonate (Arterial), Hematocrit, Hematocrit (Arterial), Hemoglobin, Hemoglobin (Arterial), International Normalized Ratio, IL6, Iron, Ketone, Lactate (Arterial), Lactate, Lactate Dehydrogenase, Lymphocyte %, Lymphocytes, Mean Corpuscular Hemoglobin Concentration, Mean Corpuscular Volume, Mean Platelet Volume, Monocyte %, Monocytes, Neutrophil %, Neutrophils, Oxygen Saturation, Oxygen Saturation (Arterial), Partial Pressure of Carbon Dioxide (Arterial), Partial Pressure of Carbon Dioxide, pH (Arterial), pH, Platelets, Partial Pressure of Oxygen (Arterial), Partial Pressure of Oxygen, Potassium, Potassium (Arterial), PT, Protein, Red Blood Cell Count, Red Cell Distribution Width, Sodium, Sodium (Arterial), Total Iron-Binding Capacity, Transferrin saturation, Troponin I, Uric Acid, Chloride (Arterial), White Blood Cell Count
